# Supplementary material for: Heparin-based hydrogel scaffolding alters the transcriptomic profile and increases the chemoresistance of MDA-MB-231 triple-negative breast cancer cells
Source: Biomater Sci. 2020 Feb 13;8(10):2786–96. doi: 10.1039/c9bm01481k (PMC7497406; doi:10.1039/c9bm01481k)
Supplement: Supplementary file 2 [file BM-008-C9BM01481K-s002.zip › Supplementary File 4/HP-B hydrogel + EGF (s)vControl/Pathways/my_analysis.Gsea.1545201170987/HALLMARK_INFLAMMATORY_RESPONSE.html]

Details for gene set HALLMARK\_INFLAMMATORY\_RESPONSE[GSEA]

|  || Dataset | expr.class.cls#HYDEGF\_versus\_CONTROL.class.cls#HYDEGF\_versus\_CONTROL\_repos |
| Phenotype | class.cls#HYDEGF\_versus\_CONTROL\_repos |
| Upregulated in class | CONTROL |
| GeneSet | HALLMARK\_INFLAMMATORY\_RESPONSE |
| Enrichment Score (ES) | -0.39861122 |
| Normalized Enrichment Score (NES) | -1.7899336 |
| Nominal p-value | 0.0 |
| FDR q-value | 0.0012125612 |
| FWER p-Value | 0.013 |
Table: GSEA Results Summary

  

Fig 1: Enrichment plot: HALLMARK\_INFLAMMATORY\_RESPONSE      
 Profile of the Running ES Score & Positions of GeneSet Members on the Rank Ordered List

  

| PROBE | DESCRIPTION (from dataset) | GENE SYMBOL | GENE\_TITLE | RANK IN GENE LIST | RANK METRIC SCORE | RUNNING ES | CORE ENRICHMENT || 1 | RASGRP1 | na |  |  | 87 | 2.987 | 0.0206 | No |
| 2 | EDN1 | na |  |  | 166 | 2.681 | 0.0391 | No |
| 3 | SCARF1 | na |  |  | 373 | 2.268 | 0.0473 | No |
| 4 | PTGER4 | na |  |  | 511 | 2.105 | 0.0579 | No |
| 5 | TNFSF9 | na |  |  | 738 | 1.875 | 0.0618 | No |
| 6 | PVR | na |  |  | 1204 | 1.566 | 0.0506 | No |
| 7 | ADORA2B | na |  |  | 1353 | 1.493 | 0.0554 | No |
| 8 | PCDH7 | na |  |  | 1559 | 1.405 | 0.0565 | No |
| 9 | RAF1 | na |  |  | 1584 | 1.399 | 0.0670 | No |
| 10 | SCN1B | na |  |  | 2225 | 1.204 | 0.0436 | No |
| 11 | GPR132 | na |  |  | 2273 | 1.188 | 0.0511 | No |
| 12 | IRF7 | na |  |  | 2931 | 1.020 | 0.0253 | No |
| 13 | F3 | na |  |  | 2957 | 1.016 | 0.0325 | No |
| 14 | SLC4A4 | na |  |  | 3004 | 1.003 | 0.0385 | No |
| 15 | GABBR1 | na |  |  | 3349 | 0.933 | 0.0283 | No |
| 16 | SLC7A1 | na |  |  | 3784 | 0.850 | 0.0127 | No |
| 17 | FZD5 | na |  |  | 4153 | 0.791 | 0.0001 | No |
| 18 | MYC | na |  |  | 4314 | 0.764 | -0.0019 | No |
| 19 | CCR7 | na |  |  | 4437 | 0.744 | -0.0020 | No |
| 20 | SLC31A2 | na |  |  | 4568 | 0.724 | -0.0027 | No |
| 21 | LIF | na |  |  | 5147 | 0.629 | -0.0277 | No |
| 22 | CHST2 | na |  |  | 5270 | 0.610 | -0.0290 | No |
| 23 | GP1BA | na |  |  | 6003 | 0.502 | -0.0632 | No |
| 24 | SPHK1 | na |  |  | 6182 | 0.478 | -0.0685 | No |
| 25 | IL7R | na |  |  | 6375 | 0.455 | -0.0747 | No |
| 26 | IL18 | na |  |  | 7482 | 0.306 | -0.1301 | No |
| 27 | SEMA4D | na |  |  | 7747 | 0.272 | -0.1417 | No |
| 28 | NLRP3 | na |  |  | 7930 | 0.252 | -0.1491 | No |
| 29 | LDLR | na |  |  | 8020 | 0.241 | -0.1518 | No |
| 30 | ATP2C1 | na |  |  | 8173 | 0.230 | -0.1578 | No |
| 31 | NFKB1 | na |  |  | 8333 | 0.205 | -0.1644 | No |
| 32 | ADRM1 | na |  |  | 8420 | 0.199 | -0.1673 | No |
| 33 | RHOG | na |  |  | 9155 | 0.108 | -0.2048 | No |
| 34 | ATP2A2 | na |  |  | 9352 | 0.081 | -0.2144 | No |
| 35 | TPBG | na |  |  | 9534 | 0.070 | -0.2233 | No |
| 36 | TNFRSF1B | na |  |  | 9814 | 0.033 | -0.2377 | No |
| 37 | BEST1 | na |  |  | 9875 | 0.025 | -0.2406 | No |
| 38 | HBEGF | na |  |  | 10340 | -0.028 | -0.2647 | No |
| 39 | IL15RA | na |  |  | 10423 | -0.038 | -0.2687 | No |
| 40 | TLR2 | na |  |  | 10870 | -0.089 | -0.2913 | No |
| 41 | SLC7A2 | na |  |  | 10928 | -0.099 | -0.2935 | No |
| 42 | IRF1 | na |  |  | 11060 | -0.115 | -0.2994 | No |
| 43 | KIF1B | na |  |  | 11259 | -0.132 | -0.3087 | No |
| 44 | RGS16 | na |  |  | 11341 | -0.139 | -0.3117 | No |
| 45 | RIPK2 | na |  |  | 11399 | -0.146 | -0.3135 | No |
| 46 | HRH1 | na |  |  | 11799 | -0.202 | -0.3327 | No |
| 47 | PTGER2 | na |  |  | 12108 | -0.234 | -0.3469 | No |
| 48 | P2RY2 | na |  |  | 12210 | -0.245 | -0.3501 | No |
| 49 | IL4R | na |  |  | 12312 | -0.263 | -0.3532 | No |
| 50 | LPAR1 | na |  |  | 12426 | -0.275 | -0.3568 | No |
| 51 | IL15 | na |  |  | 12671 | -0.309 | -0.3670 | No |
| 52 | SGMS2 | na |  |  | 12702 | -0.314 | -0.3660 | No |
| 53 | TLR3 | na |  |  | 12974 | -0.336 | -0.3774 | No |
| 54 | CXCR6 | na |  |  | 12991 | -0.339 | -0.3753 | No |
| 55 | MET | na |  |  | 13324 | -0.390 | -0.3895 | No |
| 56 | CD40 | na |  |  | 13369 | -0.398 | -0.3884 | No |
| 57 | MXD1 | na |  |  | 13508 | -0.414 | -0.3922 | No |
| 58 | ACVR2A | na |  |  | 13516 | -0.416 | -0.3890 | No |
| 59 | LAMP3 | na |  |  | 13571 | -0.423 | -0.3883 | No |
| 60 | TLR1 | na |  |  | 13598 | -0.425 | -0.3861 | No |
| 61 | KLF6 | na |  |  | 13608 | -0.426 | -0.3830 | No |
| 62 | P2RX4 | na |  |  | 13816 | -0.461 | -0.3900 | No |
| 63 | IFITM1 | na |  |  | 13940 | -0.477 | -0.3924 | No |
| 64 | SLC31A1 | na |  |  | 13988 | -0.488 | -0.3908 | No |
| 65 | GNA15 | na |  |  | 14012 | -0.490 | -0.3878 | No |
| 66 | BTG2 | na |  |  | 14115 | -0.493 | -0.3890 | No |
| 67 | RELA | na |  |  | 14142 | -0.496 | -0.3862 | No |
| 68 | PLAUR | na |  |  | 14166 | -0.503 | -0.3832 | No |
| 69 | FPR1 | na |  |  | 14461 | -0.549 | -0.3940 | Yes |
| 70 | CCL2 | na |  |  | 14503 | -0.557 | -0.3914 | Yes |
| 71 | RNF144B | na |  |  | 14528 | -0.560 | -0.3880 | Yes |
| 72 | INHBA | na |  |  | 14670 | -0.585 | -0.3905 | Yes |
| 73 | RTP4 | na |  |  | 14707 | -0.591 | -0.3874 | Yes |
| 74 | GCH1 | na |  |  | 14797 | -0.603 | -0.3870 | Yes |
| 75 | PTPRE | na |  |  | 14865 | -0.614 | -0.3853 | Yes |
| 76 | CDKN1A | na |  |  | 14882 | -0.616 | -0.3810 | Yes |
| 77 | CXCL10 | na |  |  | 14952 | -0.628 | -0.3793 | Yes |
| 78 | ATP2B1 | na |  |  | 15051 | -0.643 | -0.3790 | Yes |
| 79 | EBI3 | na |  |  | 15273 | -0.684 | -0.3849 | Yes |
| 80 | ABI1 | na |  |  | 15459 | -0.714 | -0.3886 | Yes |
| 81 | ADM | na |  |  | 15599 | -0.743 | -0.3896 | Yes |
| 82 | CX3CL1 | na |  |  | 15637 | -0.749 | -0.3852 | Yes |
| 83 | GNAI3 | na |  |  | 15764 | -0.777 | -0.3853 | Yes |
| 84 | TNFRSF9 | na |  |  | 16000 | -0.819 | -0.3907 | Yes |
| 85 | EIF2AK2 | na |  |  | 16039 | -0.830 | -0.3857 | Yes |
| 86 | ACVR1B | na |  |  | 16066 | -0.837 | -0.3800 | Yes |
| 87 | NFKBIA | na |  |  | 16276 | -0.892 | -0.3835 | Yes |
| 88 | LY6E | na |  |  | 16278 | -0.893 | -0.3760 | Yes |
| 89 | EMP3 | na |  |  | 16303 | -0.898 | -0.3697 | Yes |
| 90 | TAPBP | na |  |  | 16305 | -0.899 | -0.3622 | Yes |
| 91 | IL1A | na |  |  | 16315 | -0.902 | -0.3551 | Yes |
| 92 | SLC11A2 | na |  |  | 16327 | -0.906 | -0.3480 | Yes |
| 93 | SRI | na |  |  | 16360 | -0.913 | -0.3420 | Yes |
| 94 | LYN | na |  |  | 16413 | -0.926 | -0.3370 | Yes |
| 95 | ICOSLG | na |  |  | 16433 | -0.928 | -0.3302 | Yes |
| 96 | CD14 | na |  |  | 16500 | -0.948 | -0.3256 | Yes |
| 97 | IFNAR1 | na |  |  | 16604 | -0.970 | -0.3229 | Yes |
| 98 | TIMP1 | na |  |  | 16662 | -0.990 | -0.3175 | Yes |
| 99 | HAS2 | na |  |  | 16873 | -1.047 | -0.3197 | Yes |
| 100 | NDP | na |  |  | 16884 | -1.049 | -0.3114 | Yes |
| 101 | ICAM1 | na |  |  | 17147 | -1.127 | -0.3157 | Yes |
| 102 | CSF3 | na |  |  | 17165 | -1.132 | -0.3070 | Yes |
| 103 | PDE4B | na |  |  | 17197 | -1.143 | -0.2991 | Yes |
| 104 | ITGA5 | na |  |  | 17223 | -1.152 | -0.2907 | Yes |
| 105 | HIF1A | na |  |  | 17317 | -1.190 | -0.2855 | Yes |
| 106 | DCBLD2 | na |  |  | 17441 | -1.232 | -0.2816 | Yes |
| 107 | CMKLR1 | na |  |  | 17544 | -1.275 | -0.2762 | Yes |
| 108 | PSEN1 | na |  |  | 17614 | -1.300 | -0.2689 | Yes |
| 109 | TNFSF10 | na |  |  | 17691 | -1.328 | -0.2617 | Yes |
| 110 | CD82 | na |  |  | 17789 | -1.376 | -0.2552 | Yes |
| 111 | ABCA1 | na |  |  | 17887 | -1.432 | -0.2482 | Yes |
| 112 | ITGB8 | na |  |  | 17971 | -1.473 | -0.2402 | Yes |
| 113 | IRAK2 | na |  |  | 17984 | -1.478 | -0.2284 | Yes |
| 114 | CYBB | na |  |  | 17991 | -1.481 | -0.2162 | Yes |
| 115 | IL1R1 | na |  |  | 18099 | -1.548 | -0.2088 | Yes |
| 116 | IL1B | na |  |  | 18115 | -1.556 | -0.1965 | Yes |
| 117 | IL6 | na |  |  | 18227 | -1.619 | -0.1887 | Yes |
| 118 | NMI | na |  |  | 18304 | -1.687 | -0.1785 | Yes |
| 119 | CCL20 | na |  |  | 18309 | -1.693 | -0.1644 | Yes |
| 120 | AHR | na |  |  | 18333 | -1.702 | -0.1513 | Yes |
| 121 | IFNGR2 | na |  |  | 18361 | -1.722 | -0.1382 | Yes |
| 122 | EREG | na |  |  | 18655 | -1.994 | -0.1368 | Yes |
| 123 | OSMR | na |  |  | 18741 | -2.102 | -0.1235 | Yes |
| 124 | TNFSF15 | na |  |  | 18771 | -2.144 | -0.1070 | Yes |
| 125 | CCRL2 | na |  |  | 18863 | -2.300 | -0.0924 | Yes |
| 126 | BST2 | na |  |  | 18934 | -2.418 | -0.0758 | Yes |
| 127 | ITGB3 | na |  |  | 18981 | -2.535 | -0.0568 | Yes |
| 128 | NAMPT | na |  |  | 18985 | -2.547 | -0.0355 | Yes |
| 129 | CXCL11 | na |  |  | 19056 | -2.745 | -0.0161 | Yes |
| 130 | CD55 | na |  |  | 19064 | -2.791 | 0.0070 | Yes |
Table: GSEA details [plain text format]

  

Fig 2: HALLMARK\_INFLAMMATORY\_RESPONSE      
 Blue-Pink O' Gram in the Space of the Analyzed GeneSet

  

Fig 3: HALLMARK\_INFLAMMATORY\_RESPONSE: Random ES distribution      
 Gene set null distribution of ES for **HALLMARK\_INFLAMMATORY\_RESPONSE**
